# Supplementary material for: Dynamic transcriptomic profiles of zebrafish gills in response to zinc supplementation
Source: BMC Genomics. 2010 Oct 11;11:553. doi: 10.1186/1471-2164-11-553 (PMC3091702; doi:10.1186/1471-2164-11-553)
Supplement: Additional file 2 — Interactive Direct Interaction Network representing the molecular interactions between zinc, copper, iron, calcium and proteins encoded by transcripts changed by zinc supplementation. Mini web-site containing index.html and hyperlinked pages in subdirectory describing a Direct Interaction Network automatically generated based on curated interactions contained within the proprietary PathwayArchitect database. Ovals represent proteins and the circles symbolize metal ions. Objects are coloured by their abundance in zebrafish at the time-point they were significantly different from the control is a scale from -4 fold (dark green) to +4 fold (dark red). Where significant differences were found at more than one time-point, the colour overlay shows expression at the first instance. Dark blue squares denote 'binding', and light blue squares 'expression'; green squares stand for 'regulation', green diamonds for 'metabolism', and green circles for 'promoter binding'. Arrow heads indicate directionality of the interaction where annotated. All nodes and edges can be further interrogated by selecting the relative area of the image. [file 1471-2164-11-553-S2.zip › PathwayArchitect Zn xs DIN/99982.html]

# PROTEIN: MFI2

|  |  |
| --- | --- |
| Name | MFI2 |
| Type | PROTEIN |
| Description | antigen p97 (melanoma associated) identified by monoclonal antibodies 133.2 and 96.5 |
| Note | The protein encoded by this gene is a cell-surface glycoprotein found on melanoma cells. The protein shares sequence similarity and iron-binding properties with members of the transferrin superfamily. The importance of the iron binding function has not yet been identified. This gene resides in the same region of chromosome 3 as members of the transferrin superfamily. Alternative splicing results in two transcript variants. |
| Alias | MFI2 |
|  | CD228 |
|  | melanotransferrin |
|  | MTF1 |
|  | Mtf |
|  | Mfi2 |
|  | Melanoma-associated antigen p97 |
|  | MTf |
|  | Membrane-bound transferrin-like protein p97 |
|  | melanoma-associated antigen p97 |
|  | MGC4856 |
|  | MAP97 |
|  | CD228 antigen |


---

|  |  |
| --- | --- |
| GO Component | extracellular space |
|  | extracellular region |
|  | integral to plasma membrane |
|  | membrane |


---

|  |  |
| --- | --- |
| GO ID | GO:0006879 |
|  | GO:0006810 |
|  | GO:0016020 |
|  | GO:0005615 |
|  | GO:0005506 |
|  | GO:0008199 |
|  | GO:0046872 |
|  | GO:0005887 |
|  | GO:0006826 |
|  | GO:0005576 |


---

|  |  |
| --- | --- |
| MIM | MIM:155750 |


---

|  |  |
| --- | --- |
| Connectivity | 36 |


---

|  |  |
| --- | --- |
| Entrez ID | 30060 |
|  | 4241 |


---

|  |  |
| --- | --- |
| Agilent ID | A\_23\_P212042 |
|  | A\_53\_P108278 |
|  | A\_51\_P324351 |
|  | A\_23\_P386320 |
|  | A\_14\_P102948 |
|  | A\_53\_P136221 |


---

|  |  |
| --- | --- |
| Cellular Localization | Membrane |
|  | Extracellular region |
|  | Cell |
|  | Plasma membrane |


---

|  |  |
| --- | --- |
| Pathway | Zn def RIN |
|  | Zn xs inventory |
|  | Zn xs DIN |


---

|  |  |
| --- | --- |
| GO Process | transport |
|  | iron ion transport |
|  | iron ion homeostasis |


---

|  |  |
| --- | --- |
| UniGene | Hs.184727 |
|  | Mm.30988 |


---

|  |  |
| --- | --- |
| Affymetrix Probeset ID | 1556538\_at |
|  | 220043\_s\_at |
|  | 223723\_at |
|  | 1419755\_at |
|  | 83377\_at |
|  | aa674893\_at |
|  | g12804858\_3p\_at |
|  | g5729923\_3p\_s\_at |
|  | Hs2.348653.1.S1\_3p\_at |
|  | Msa.25882.0\_s\_at |
|  | 235911\_at |
|  | 239521\_at |
|  | 242087\_x\_at |
|  | 243629\_x\_at |
|  | 76563\_at |
|  | 88946\_at |
|  | Hs.128852.0.A1\_3p\_a\_at |
|  | Hs.128852.0.A1\_3p\_at |
|  | Hs.128852.1.A1\_3p\_x\_at |
|  | Hs.128852.2.A1\_3p\_x\_at |
|  | 85279\_at |
|  | 97970\_at |
|  | Hs.184727.0.A1\_3p\_at |
|  | Msa.6814.0\_s\_at |
|  | TC18213\_at |


---

|  |  |
| --- | --- |
| GO Function | ferric iron binding |
|  | iron ion binding |
|  | metal ion binding |


---

|  |  |
| --- | --- |
| Nucleotide | AB024336 |
|  | BC002623 |
|  | M12154 |
|  | AK036444 |
|  | BC040347 |
|  | BC007550 |
|  | AB047799 |
|  | BC093920 |
|  | A00127 |
|  | NM\_005929 |
|  | BC071910 |
|  | BC001875 |
|  | BC053510 |
|  | NM\_033316 |
|  | NM\_013900 |
|  | BC032752 |
|  | BT007377 |


---

|  |  |
| --- | --- |
| Protein | NP\_005920 |
|  | NP\_201573 |
|  | AAP36041 |
|  | P08582 |
|  | CAA00012 |
|  | AAH07550 |
|  | BAB41139 |
|  | BAA86655 |
|  | AAH02623 |
|  | AAH71910 |
|  | AAA59992 |
|  | AAH40347 |
|  | NP\_038928 |
|  | AAH93920 |
|  | Q9R0R1 |
|  | AAH01875 |
|  | BAC29432 |


---

|  |  |
| --- | --- |
| Organism | Mammal |


---

|  |  |
| --- | --- |
| Location | chromosome 3, 3q28-q29 (Homo sapiens) |
|  | chromosome 16, 16 B3 (Mus musculus) |


---

|  |  |
| --- | --- |
